# Determination of the Relationship of Serum Hyaluronic Acid Levels to the Degree of Liver Fibrosis in Biopsies of Patients with Chronic Viral Hepatitis B and C   

**Published:** 2010-09-01

**Authors:** Faride Moradi Moghaddam, Hossein Arrbabi, Mohammad Khajedaloei

**Affiliations:** 1 Department of Internal Medicine, Ghaem Hospital, Mashhad University of Medical Sciences, Mashhad, Iran; 2 Department of Public Medicine, Ghaem Hospital, Mashad University of Medical Sciences, Mashhad, Iran

**Keywords:** Cirrhosis, Hyaluronic Acid, Chronic Viral Hepatitis

## Abstract

**Background and Aims:**

Determining the stage of liver fibrosis and the grade of necroinflammation is important in predicting the prognosis and treatment response of patients with chronic viral hepatitis. Liver biopsy is associated with several technical problems and complications; therefore, its repetitive performance as a procedure in evaluating treatment response and in following up patients is not recommended. This study was performed to determine the correlation of serum hyaluronic acid (HA) levels with the stage and grade of histopathologic liver changes in patients with chronic hepatitis B and C.

**Methods:**

This analytical cross-sectional study was performed on 69 patients with chronic hepatitis B or C in Mashhad, Iran, who were referred to our clinic and underwent liver biopsy and blood sampling simultaneously in 2006-2007. None of the patients were on medication that would affect the serum levels of (HA) and patients with signs of arthropathy were excluded from the study.

**Results:**

Out of the 69 patients i  this study, 48 (69.6%) were male and 21 (30.4%) were female. The causative virus in 29 patients (42%) was hepatitis C and in the rest (n = 40, 58%) hepatitis B. The severity of liver fibrosis (stage) had a direct correlation (r = 0.25, P = 0.042) with the levels of (HA) and an inverse correlation with the platelet level (r = 0.37, P = 0.002). The degree of liver necroinflammation (grade) did not have a significant correlation with the levels of (HA) and alanine transaminase (r = -0.01, P = 0.9; r = 0.21, P = 0.09 respectively); instead, there was an inverse correlation with platelet levels (r = 0.36, P = 0.003).

**Conclusions:**

Our study results correspond with those of other international studies; thus suggesting that the (HA) serum level is a useful marker in determining the severity of fibrosis in patients with chronic viral hepatitis. To form a definite conclusion, further studies on large groups should be performed.

## Introduction

Chronic injury to the liver leads to its fibrosis [1]. Hepatitis B and C are examples of viral infections that cause chronic hepatitis; even asymptomatic patients show some degree of liver fibrosis (LF) that can range from mild to end-stage cirrhosis [2][3][4]. Determining the degree of LF in chronic viral hepatitis is necessary in determining a patient’s prognosis and indications for therapy. Although liver biopsy is a gold- standard procedure in determining the stage and grade of LF [3][5][6][7], it can be associated with complications (e.g. bleeding and pain at the biopsy site, and a prolonged hospital stay), high hospital expenses [3][8], false sample recording [9][10][11], and in some cases contraindications during the procedure, and dependence on pathologists’ skills in examining samples. Considering the aggressiveness of the procedure, repeating it for further evaluation and follow-up to determine treatment response is not recommended [6][8]. From a clinical perspective, the use of a non-invasive procedure in diagnosing and determining the presence and degree of LF, and as a tool in following up on the effects of anti-viral and antifibrotic medicines on the liver [8][12][13] is needed. LF is a complex and dynamic process with an increased extracellular matrix, increased activity of the matrixproducing cells, release of cytokines and, ultimately, structural tissue changes [3]. Hyaluronic acid (HA) is a polysaccharide with a high molecular weight found in the extracellular spaces. Through the bloodstream, it enters the lymphatic system where it is quickly degraded and removed by the sinusoidal endothelial cells [3][14]. Recent studies have shown that HA levels are elevated in patients with LF [1][2][3][4][6][14][15][16]. This study aimed to compare HA serum levels in different stages and grades (semi-quantitatively) in liver biopsies of patients with chronic hepatitis B and C.

## Materials and Methods

This analytical cross-sectional study was done on patients with chronic hepatitis B or C, who were referred to the Gastrointestinal and Liver Disease Research Center of Imam Reza Hospital in Mashhad from 2006 to 2007. All of the patients involved in the study were selected from a census of their medical files, and a liver biopsy and a blood sample were concurrently done.

Those patients who did not have a liver biopsy done concurrently with a blood test were not enrolled in the study.

The population under study included 40 patients with chronic hepatitis B, and 29 patients with chronic hepatitis C, all with available serum samples. None of the patients used medicine that affected HA levels, at the time of the study. Considering that HA synthesis also occurs in the synovial membranes and that there is increased joint inflammation in these patients, those with signs of clinical arthropathy were excluded from the study. Also, those with a history of daily use of vitamin A and/or amiodarone and methotrexate causing LF were excluded from the study. The HA test kit was provided by Corgenix Inc. (Colorado, USA, under license of Chugai Diagnostic Science Co.), and the HA serum levels were measured quantitatively by the enzyme-linked immunosorbent assay (ELISA) according to the manufacturer’s instructions (normal value = 0-75 µg/l). The results were then compared with the findings from the liver biopsies.

All liver biopsies were studied by histopathologic methods, using a trichromic stain that showed fibrosis. The degree of LF was determined based on Knodell’s scoring system. Slides were studied by experienced pathologists and findings classified into three groups, mild (stages 0,1,2), moderate (stages 3,4) and severe (stages 5,6). The degree of liver tissue inflammation and necrosis was also determined and classified according to Knodell’s scoring system, mild (grades 0-4), moderate (grades 5-8), relatively severe (grades 9-12) and severe (grades 13-18). None of the pathologists were aware of the patients’ serum HA levels.

Data analysis was done with the software program SPSS version 13. First, the demographic findings, distributive and central indices of the patients were analyzed by the descriptive statistical method and appropriate charts were used. Chi-square tests were used to compare qualitative variables, and the t-test was used for quantitative variables. Non-parametric tests were used for non-Gaussian distributive patterns.

Patients’ blood sample results were retrieved from the archives of the blood bank of the Liver Research Center of the University of Mashhad. Patients were provided with information about the research and their consent obtained before the procedures were carried out. Patients’ details were kept confidential by the researchers.

## Results

Out of the 69 patients studied, 48 (69.6%) were men and 21 (30.4%) women. The mean age of the patients was 34 ± 10. The youngest patient was 15 years old and the oldest 61. Twenty-nine (42%) patients had chronic hepatitis C, while the rest (40 = 58%) had chronic hepatitis B. The demographic and paraclinical findings of these patients, based on the viral agent for their chronic disease, are shown in Table 1. There was no significant difference in the mean age, number of platelets, alanine transaminase (ALT) and HA levels between the two groups (chronic hepatitis B and C). The mean levels of HA, ALT and number of platelets were 54.48±25.34 µg/l, 87.91 ±71.4 µg/l and 175.92 ± 49.9×103/ml respectively. The mean of these variables based on the degree of LF (stage) and degree of liver necroinflammation (grade) is shown in Table 2 and Table 3.Spearman’s statistical test showed a significant, direct correlation between the severity of liver fibrosis (stage) and the level of HA (r = 0.25, P = 0.042) and a significant inverse correlation with the number of platelets (r= -0.37, P = 0.002), but there was no significant correlation with the levels of serum ALT (r = 0.36, P = 0.44). Spearman’s rho test also showed a significant inverse correlation between the degree of inflammation (grade) and the number of platelets (r = -0.36, P = 0.003), but no significant correlation between the degree of inflammation, HA (r = 0.01, P = 0.99) and ALT levels (r = 0.21, P = 0.09).

**Table 1:** Demographic and paraclinical findings of the study population^a^

**Variable**		**Type of virus**		**P-value**
		**Hepatitis B**	**Hepatitis C**	
Gender	Male	26	22	-
	Female	7	14	
Age		36.62 ±10.25	31.77 ± 10.28	0.06
Platelets		168000±49000	181000±49000	0.22
ALT		101.03±95.4	78.82±47.39	0.86
HA		56.17±25.51	53.25 ±25.48	0.42

^a^ : HA: Hyaluronic acid; ALT: Alanine aminotransferase

**Table 2:** The mean of HA, platelet and ALT levels based on the degree of liver fibrosis (stage).^a^

**stage**	**ALT**	**Variable Platelet( ×10^3^)**	**HA**
Mild (0,1 and 2)	88.33±79.52	187.31±51.32	50.5±21.57
Moderate (3 and 4)	77.75±52.09	161.41±42.74	57.92±24.91
Severe (5 and 6)	100.88±59.04	136.67±32.35	71.89±38.85
Statistical Result	r = 0.36 , P = 0.44	r = -0.37 , P = 0/002	r = 0.25 , P = 0.042

^a^ : HA: Hyaluronic acid; ALT: Alanine aminotransferase

**Table 3:** The mean of serum HA, platelets and ALT levels based on the degree of liver inflammation and necrosis (Grade).^a^

**Grade**	**HA**	**Variable Platelet (×10^3^)**	**ALT**
Mild (0-4)	54.91±24.38	194.71±38.5	87.62±80.34
Moderate (5-8)	52.94±21.84	175.03±58.48	67.42±35.65
Relatively severe (12-18)	59.5±37.13	147.09±30.11	139.91±106.1
Severe (13-19)	45±14.14	166.5±13.43	117±5.65
Statistical Result	r = 0.01 ,P=0.99	r = -0.36 , P=0.003	r = 0.021 ,P=0.09

^a^ : HA: Hyaluronic acid; ALT: Alanine aminotransferase

**Figure 1:**
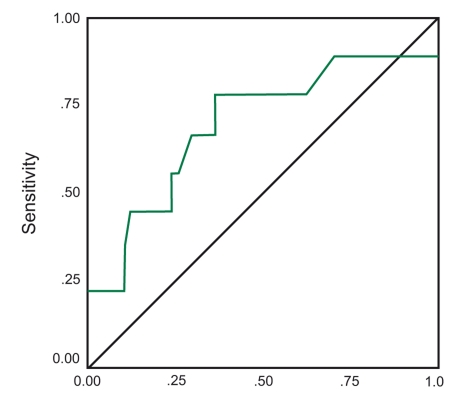
Sensitivity and specificity of the hyaluronic acid test in the distinction of different stages of liver fibrosis (area = 0.693)

**Figure 2:**
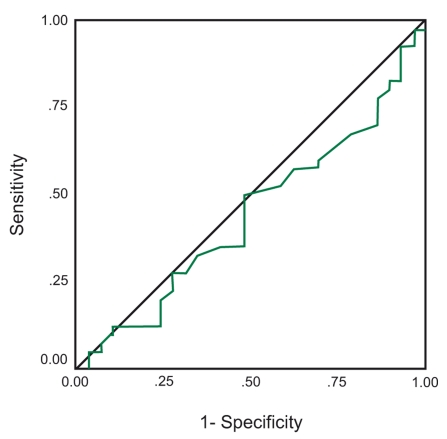
Sensitivity and specificity of the hyaluronic acid test in the diagnosis of liver fibrosis (area = 0.443).

Calculation of the area under receiver operating characteristic (ROC) curves showed (Fig 1) that the sensitivity and the specificity of the HA test was desirable in distinguishing different stages of liver fibrosis (area = .693), but was not suitable (Fig 2) for diagnosing these histopathologic liver changes (area=0.443).

## Discussion

A wide spectrum of chronic liver diseases can cause LF. Fibrosis leads to the collapse of hepatic lobules, produces fibrotic walls and causes the degeneration of hepatocytes, altering the liver to a nodular tissue. Liver biopsy shows these histopathologic changes, determines the degree of fibrosis and is a guide to treatment decisions. Because regular serological and biochemical tests have little value in determining the severity of inflammation and fibrosis, biopsy still remains the gold-standard procedure. However, liver biopsy has its limitations, it is an invasive procedure associated with complications, and its interpretation depends on pathologists’ skills. Several studies have used different markers, mostly of extracellular matrix origin, to determine the degree of LF in patients with different chronic liver conditions. The accumulation of extracellular matrix in the liver [16][17][18][19][20] occurs due to an imbalance in production and degradation. At the same time, these changes are accompanied by portal hypertension and abnormal liver function [13][20]. HA is a vital component in producing viscoelasticity in the extracellular matrix; it also lubricates the interstitial tissue. HA is synthesized in the plasma membrane of fibroblasts and other cells, especially the connective tissue and synovial membrane. A small percentage of HA is locally metabolized while the greater part of it enters the blood through the lymphatic system and from there goes into the liver where it is immediately metabolized by the hepatoendothelial cells [17][18]. In a healthy individual, HA spends half of its life in the blood stream (2-5 minutes). Its normal serum concentration is age-dependent. From the neonatal stage to puberty and then during adulthood, its concentration usually rises. Any condition that interferes with the removal of HA from the blood into the endothelial cells of the liver will cause its level to become elevated.

In this study, the relationship between serum HA levels and the degree of LF in patients with hepatitis B and C  was determined. Our findings have shown similarities to those of previous studies done on this topic. Nyberg et al. [21] have shown that HA levels are a sensitive tool that can be used to determine the degree of progressive liver injury in patients with primary biliary cirrhosis. Another study by Fried [18] showed that patients who developed LF from chronic veno-occlusive diseases also presented with elevated HA levels. Other similar studies involving patients with hepatitis C also showed a direct correlation between fibrosis in liver biopsies and elevated HA levels, which completely corresponds to the findings of our recent study [7][9][10][19]. Our results were also similar to the studies of Wong and Skirpenova [9][19], where no significant correlation between the degree of inflammation and HA and serum ALT levels was found. Although this study showed an inverse correlation between the severity of fibrosis and the number of platelets, this had never been studied before in prior research. Wyatt et al. had studied children with fibrocystic diseases and discovered that patients who developed LF had a reasonable elevation in serum HA levels, compared to those who did not have fibrosis [17]. Some other researchers have found similar results in their studies of chronic liver disease in both children and adults [5][16]. In Iran, Montazeri et al., (2005) also reported a relationship between serum hyaluronate and the severity of inflammation and fibrosis in patients with non-HBeAg hepatitis B [6].

## Conclusions

The correlation of the results in our study with similar studies worldwide proves the theory that measuring serum levels of HA is a useful tool in determining the severity of fibrosis in patients with hepatitis B and C and is a suitable non-invasive procedure that can replace invasive liver biopsy. Nevertheless, further studies on a large group of patients should be performed before reaching a definite conclusion.

## References

[1] Zheng M, Cai WM, Weng HL, Liu RH (2002). ROC curves in evaluation of serum fibrosis indices for hepatic fibrosis. World J Gastroenterol.

[2] Pungpapong S, Kim WR, Poterucha JJ (2007). Natural history of hepatitis B virus infection: an update for clinicians. Mayo Clin Proc.

[3] Khan JA, Khan FA, Dilawar M, Ijaz A, Khan NA, Mehmood T (2007). Serum hyaluronic acid as a marker of hepatic fibrosis. J Coll Physicians Surg Pak.

[4] Yilmaz S, Bayan K, Tüzün Y, Dursun M, Kaplan A, Ozmen S, Canoruç F, Akkuş Z (2007). Replacement of hystological findings: serum hyaluronic acid for fibrosis, high-sensitive C-reactive protein for necroinflamation in chronic viral hepatitis. Int J Clin Pract.

[5] Lu LG, Zeng MD, Wan MB, Li CZ, Mao YM, Li JQ, Qiu DK, Cao AP, Ye J, Cai X, Chen CW, Wang JY, Wu SM, Zhu JS, Zhou XQ (2003). Grading and staging of hepatic fibrosis, and its relationship with noninvasive diagnostic parameters. World J Gastroenterol.

[6] Montazeri G, Estakhri A, Mohamadnejad M, Nouri N, Montazeri F, Mohammadkani A, Derakhshan MH, Zamani F, Samiee S, Malekzadeh R (2005). Serum hyaluronate as a non-invasive marker of hepatic fibrosis and inflammation in HBeAg-negative chronic hepatitis B. BMC Gastroenterol.

[7] Halfon P, Bourlière M, Pénaranda G, Deydier R, Renou C, Botta Fridlund D, Tran A, Portal I, Allemand I, Rosenthal Allieri A, Ouzan D (2005). Accuracy of hyaluronic acid level for predicting liver fibrosis stages in patients with hepatitis C virus. Comp Hepatol.

[8] Lebensztejn DM, Skiba E, Tobolczyk J, Sobaniec Lotowska ME, Kaczmarski M (2005). Diagnostic accuracy of serum biochemical fibrosis markers in children with chronic hepatitis B evaluated by receiver operating characteristics analysis. World J Gastroenterol.

[9] Skripenova S, Trainer TD, Krawitt EL, Blaszyk H (2007). Variability of grade and stage in simultaneous paired liver biopsies in patients with hepatitis C. J Clin Pathol.

[10] Regev A, Berho M, Jeffers LJ, Milikowski C, Molina EG, Pyrsopoulos NT, Feng ZZ, Reddy KR, Schiff ER (2002). Sampling error and intraobserver variation in liver biopsy in patients with chronic HCV infection. Am J Gastroenterol.

[11] Colloredo G, Guido M, Sonzogni A, Leandro G (2003). Impact of liver biopsy size on histological evaluation of chronic viral hepatitis: the smaller the sample, the milder the disease. J Hepatol.

[12] Guechot J, Serfaty L, Bonnand AM, Chazouilleres O, Poupon RE, Poupon R (2000). Prognostic value of serum hyaluronan in patients with compensated HCV cirrhosis. J Hepatol.

[13] Vrochides D, Papanikolaou V, Pertoft H, Antoniades AA, Heldin P (1996). Biosynthesis and degradation of hyaluronan by nonparenchymal liver cells during liver regeneration. Hepatology.

[14] Hartley JL, Brown RM, Tybulewicz A, Hayes P, Wilson DC, Gillett P, McKiernan P (2006). Hyaluronic acid predicts hepatic fibrosis in children with hepatic disease. J Pediatr Gastroenterol Nutr.

[15] Kim MY, Baik SK, Jang YO, Suk KT, Kim JW, Kim HS, Cho MY, Choi SJ, Um SH, Han KH (2008). Serum hyaluronic acid level: correlation with quantitative measurement of hepatic fibrosis in a cirrhotic rat model. Korean J Hepatol.

[16] Ding H, Chen Y, Feng X, Liu D, Wu A, Zhang L (2001). Correlation between liver fibrosis stage and serum liver fibrosis markers in patients with chronic hepatitis B. Zhonghua Gan Zang Bing Za Zhi.

[17] Wyatt HA, Dhawan A, Cheeseman P, Mieli Vergani G, Price JF (2002). Serum hyaluronic acid concentrations are increased in cystic fibrosis patients with liver disease. Arch Dis Child.

[18] Fried MW, Duncan A, Soroka S, Connaghan DG, Farrand A, Peter J, Strauss RM, Boyer TD, McDonald GB (2001). Serum hyaluronic acid in patients with veno-occlusive disease following bone marrow transplantation. Bone Marrow Transplant.

[19] Wong VS, Hughes V, Trull A, Wight DG, Petrik J, Alexander GJ (1998). Serum hyaluronic acid is a useful marker of liver fibrosis in chronic hepatitis C virus infection. J Viral Hepat.

[20] Curry M, Afdhal H (2005). Serum markers of hepatic fibrosis.. UpToDate.

[21] Nyberg A, Engstrom Laurent A, Loof L (1988). Serum hyaluronate in primary biliary cirrhosis--a biochemical marker for progressive liver damage. Hepatology.

